# Evaluating the Antimicrobial Efficacy of Citral Nano-Emulsion Against *Vibrio parahaemolyticus*

**DOI:** 10.3390/foods14183272

**Published:** 2025-09-21

**Authors:** Juanjuan Cao, Xiaoxu Zhang, Zihe Qi, Huan Liu

**Affiliations:** 1 School of Food Science and Engineering, Shaanxi University of Science & Technology, No. 6 Xuefu Road, Xi’an 710021, China; 19829264920@163.com (J.C.); 201604020228@sust.edu.cn (X.Z.); zhq_lucky@163.com (Z.Q.); 2 Shaanxi Research Institute of Agriculture Products Processing Technology, No. 6 Xuefu Road, Xi’an 710021, China

**Keywords:** citral nano-emulsion, *Vibrio parahaemolyticus*, antimicrobial activity, salmon preservation

## Abstract

Citral is extensively utilized in the realm of food preservation owing to its excellent antibacterial activity. Nevertheless, being a common essential oil, citral’s hydrophobic characteristic considerably limits its potential use and marketability. In this study, we prepared hydrophobic citral into an oil-in-water nano-emulsion by high-pressure homogenization to address its solubility issues in water. The optical ratio of the citral nano-emulsion was established using a combination of response surface experiments. Subsequently, the citral nano-emulsion was employed to suppress *Vibrio parahaemolyticus* RIMD 2210633 (*V. parahaemolyticus*). The findings indicated that the citral nano-emulsion had a minimum inhibitory concentration (MIC) of 0.125 mg/mL and a minimum bactericidal concentration (MBC) of 0.25 mg/mL against *V. parahaemolyticus*, respectively. Furthermore, the nano-emulsion displayed excellent antibacterial properties, mainly by causing cell envelope damage, and also inhibited the production of virulence factors. Finally, citral nano-emulsion was applied to salmon preservation and efficiently controlled the propagation of *V. parahaemolyticus* in salmon. Our research has addressed the limitations associated with the application of citral and expanded the applications for its use in the food industry.

## 1. Introduction

With the exacerbation of antibiotic resistance, there is an urgent need to develop green, efficient, and less vulnerable alternatives to bacterial resistance. Fortunately, the majority of plant-derived compounds exhibit a variety of biological activities, making them a great choice for generating novel antibacterial agents [1]. Plant-derived antibacterial agents are in great demand and have a wide range of uses in the food industry due to their natural and excellent safety properties [2]. These compounds come in a variety of forms and are widely available, mostly from hydrophobic secondary metabolites found in plants [3,4]. Among them, numerous essential oils derived from diverse plants or herbs exhibit strong antibacterial capabilities.

Citral is a common aliphatic aldehyde naturally found in various citrus essential oils [5]. In addition to being abundant in lemongrass oil, it is also widely present in the leaves and fruits of limes, oranges, lemons, tomatoes, myrtle trees, and African basil [6]. The United States Food and Drug Administration recognizes and acknowledges citral as a safe food additive [7]. Furthermore, citral was extensively utilized for food preservation due to its outstanding antibacterial properties [8,9]. Research showed that the synergistic antibacterial effect of citral and eugenol can extend the shelf life of bread [10]. Not only that, several studies have demonstrated that citral efficiently suppresses the growth and reproduction of pathogenic and degrading microorganisms, extending the shelf life of foods such as meat products, fish, fruits, vegetable juice, and bread [11,12,13,14,15,16]. The application of citral in food preservation is becoming increasingly popular. However, its poor water solubility and volatility limit its application in water-soluble foods and other applications. Nano-emulsion development effectively addresses citral’s water insolubility, introducing a novel avenue for the broader utilization of this compound.

Nano-emulsions are a class of emulsions with droplet sizes ranging from 20 nm to 100 nm, and oil-in-water nano-emulsions are crucial carriers of water-insoluble antimicrobial agents [17,18]. Because of the small droplet size, nano-emulsions are more stable than conventional emulsions in terms of emulsification, aggregation, flocculation, and Ostwald ripening [19]. Droplet size is a critical determinant of emulsion stability and release kinetics [20]. By optimizing the process parameters, the nano-emulsion system with small particle size and higher stability can be prepared for the application of antibacterial agents. Nanoscale emulsion droplets interact with and fuse into the cellular membranes of bacteria or the envelopes of viruses, consequently destabilizing their lipid structures and facilitating the leakage of internal cellular contents [21]. In addition, this non-specific antimicrobial mode of action does not lead to the development of drug-resistant microbial strains [22]. Recent years have witnessed significant advances in the applied research of nano-emulsions, with applications spanning pharmaceuticals, food science, cosmetics, and industrial processes.

In this present study, the preparation conditions for citral nano-emulsion were first determined using single-factor experiment and response surface tests. The citral nano-emulsion were characterized by determining the particle size, polydispersity index (PDI), Fourier transform infrared spectroscopy (FTIR), pH, and turbidity. The bacteriostatic and attenuated activities of citral nano-emulsion against *Vibrio parahaemolyticus* RIMD 2210633 (*V. parahaemolyticus*) were determined. *V. parahaemolyticus* is a foodborne pathogen widely present in marine environments, which can be transmitted through raw seafood and cause symptoms such as diarrhea, vomiting, and abdominal pain in humans. The massive pollution of seafood products has caused huge losses to human safety and the economy. Hence, the inhibitory effect of citral nano-emulsion on *V. parahaemolyticus* was evaluated in salmon, providing a foundation for its development and application as a potential antimicrobial agent against *V. parahaemolyticus*.

## 2. Materials and Methods

### 2.1. Reagents

Citral (Shanghai Yuanye Biotechnology Co., Ltd., Shanghai, China), Tween 80 and NaCl (Tianjin Tianli Chemical Reagent Co., Ltd., Tianjin, China), Tryptone and Yeast Extract (Thermo Fisher Scientific, Waltham, MA, USA), glutaraldehyde (Tianjin Kemiou Chemical Reagent Co., Ltd., Tianjin, China), isoamyl acetate (Fuchen Chemical Reagent Co., Ltd., Tianjin, China), Glucose detection kit and Malondialdehyde (MDA) test kit (Nanjing Jiancheng Biotechnology Co., Ltd., Nanjing, China), ethyl alcohol absolute (Sangon Biotech Shanghai Co., Ltd., Shanghai, China), deionized water (Millipore, Billerica, MA, USA), KBr (Tianjin Kemiou Chemical Reagent Co., Ltd., Tianjin, China), alkaline phosphatase (AKP) Kit (Shanghai Beyotime Biotechnology Co., Ltd., Shanghai, China), Thiosulfate citrate bile sucrose (Qingdao Hope Biotechnology Co., Ltd., Qingdao, China), Ampicillin (Beijing Biotopped Technology Co., Ltd., Beijing, China) were used.

### 2.2. Strain and Culture Conditions

*V. parahaemolyticus* RIMD 2210633 was purchased from the China General Microbiological Culture Collection Center (CGMCC). The strain was stored in a glycerine tube for a long time (−80 °C). During the experiment, Luria–Bertani (LB) liquid medium (1% NaCl, 1% tryptone, and 0.5% yeast extract) was used to propagate at 37 °C with the oscillation frequency of 200 r/min in the whole study unless stated otherwise.

### 2.3. Preparation of Citral Nano-Emulsion

Citral nano-emulsions were prepared via high-pressure homogenization using a homogenizer (AH100B, Antos Nano Technology Co.,Ltd., Suzhou, China). Briefly, a predetermined amount of citral (e.g., 8% *v*/*v*) and Tween 80 (e.g., 10% *v*/*v*) were added to distilled water under continuous mixing. A coarse emulsion was first formed using a magnetic stirrer operating at 900 rpm for 10 min at room temperature (e.g., 25 °C). This pre-emulsion was then subjected to high-pressure homogenization. The homogenization process was carried out at a pressure of (e.g., 800 bar) for three consecutive cycles. The final nano-emulsion was stored in amber vials for further characterization.

### 2.4. Experiment Design

Based on preliminary single-factor experiments, the central points for the response surface design were determined. A citral concentration of 8% (*v*/*v*), a Tween 80 concentration of 10% (*v*/*v*), and 800 bar were selected, as this combination yielded nano-emulsions with smaller droplet size, lower PDI, and improved stability compared to other tested ratios. Using these central values, a three-factor (X1, X2, and X3), three-level Box–Behnken design was employed to optimize the droplet size. The basic specifications of the nano-emulsion are shown in Table 1. The optimized formula was obtained by response surface design to prepare citral nano-emulsion. Then the particle size and PDI of the citral nano-emulsion were measured.

### 2.5. Characterization of Citral Nano-Emulsion

To comprehensively characterize the physicochemical properties of the prepared citral nano-emulsion, multiple analytical techniques were employed, including measurements of particle size, PDI, FTIR, pH, turbidity, and stability over time. The mean particle size and PDI of the nano-emulsion were determined using a Zeta Sizer (Zetasizer Nano ZS90, Malvern Instruments Ltd., Malvern, UK). Specifically, the sample was diluted 200-fold with deionized water until optically clear and transferred into a 1 mL quartz cuvette for measurement. Each measurement was performed in triplicate. FTIR (VECTOR-22 Model 680, Bruker Optics, Ettlingen, Germany) was used to characterize the presence of functional groups in the citral nano-emulsion. Spectra were acquired using the liquid film method: one drop of the nano-emulsion was applied onto a pre-pressed KBr pellet to form a uniform thin film. The sample was then mounted in the holder and scanned within the range of 4000–400 cm^−1^. The pH of the nano-emulsion was monitored every other day over a period of 14 days using a calibrated pH meter (PHS-3C, Shanghai Yidian Scientific Instrument Co., Ltd., Shanghai, China). Prior to measurements, the pH meter was calibrated with standard buffer solutions at pH 4.00, 6.86, and 9.18. The electrode was thoroughly rinsed with deionized water between replicates, and three independent measurements were taken per time point, with the results reported as mean ± standard deviation. Turbidity was assessed by measuring the absorbance at 600 nm using ultraviolet spectrophotometer (SP-756P, Shanghai Spectrum Instruments Co., Ltd., Shanghai, China). The nano-emulsion was diluted 10-fold with deionized water (which served as the blank control) prior to measurement. The turbidity calculation formula was as follows:

(1) T = 2.302*A V*/*I*

*A*—absorbance of the diluted nano-emulsion at 600 nm;

*V*—dilution ratio;

*I*—optical path difference 0.01 m.

The stability of citral nano-emulsion was monitored by the changes in particle size and PDI at 0, 7, and 14 days, respectively.

### 2.6. Determination of Minimum Inhibitory Concentration (MIC) and Minimum Bactericidal Concentration (MBC)

The MIC of the citral nano-emulsion against *V. parahaemolyticus* was determined using a two-fold broth dilution method. Briefly, the nano-emulsion was added to LB liquid medium to achieve final citral concentrations (calculated based on the actual citral content) of 2, 1, 0.5, 0.25, 0.125, 0.0625, 0.03125, and 0 mg/mL. Each medium was then inoculated with 1% (*v*/*v*) of an overnight culture of *V. parahaemolyticus* and incubated at 37 °C with shaking at 200 rpm for 12 h. Bacterial growth was assessed visually. The MIC was defined as the minimum concentration of citral nano-emulsion that completely inhibited the visible growth of *V. parahaemolyticus*. Additionally, bacterial growth was monitored by measuring the optical density at 600 nm at hourly intervals to generate growth curves. For the MBC assay, 50 μL aliquots from cultures treated with 4MIC, 2MIC, MIC, and 1/2MIC of the nano-emulsion were spread onto Thiosulfate Citrate Bile Sucrose (TCBS) agar plates. After spreading evenly, the plates were inverted and incubated at 37 °C for 24 h. The MBC was defined as the lowest concentration that resulted in no colony growth, corresponding to ≥99.9% killing efficiency.

### 2.7. Determination of AKP Content

The activated *V. parahaemolyticus* was inoculated into LB medium and cultured for 3 h to make OD_600 nm_ reach 0.5. The nano-emulsion was added so that the final concentration of the solution was 0, 1/4MIC, 1/2MIC, and MIC, respectively. The mixed solution was incubated in an incubator at 37 °C, 200 r/min for 10 h, and the samples were taken every two hours. After centrifugation at 4000 r/min for 3 min, the content of AKP in the supernatant was determined according to the AKP detection kit.

### 2.8. MDA Content Assay

The contents of MDA in the supernatants were determined according to the instructions in the lipid peroxidation MDA assay kit. Sample pretreatment was as follows: The activated *V. parahaemolyticus* was inoculated into LB medium and cultured for 3 h (OD_600 nm_ = 0.5). The nano-emulsion was added to achieve final concentration of 0, 1/4MIC, 1/2MIC, and MIC, respectively. Samples were taken every 2 h, centrifuged at 4000 r/min for 3 min, and the MDA content was measured at 532 nm optical density.

### 2.9. Extracellular Glucose Content Assay

*V. parahaemolyticus* was cultured for 3 h until OD_600 nm_ = 0.5 and inoculated in the solution of LB broth containing different concentrations of citral nano-emulsion (0, 1/4MIC, 1/2MIC, and MIC), and shaking for 10 h at 200 r/min at 37 °C. Samples were collected at two-hour intervals, and glucose content was quantified in accordance with the protocol provided with the glucose assay kit.

### 2.10. Field Emission Scanning Electron Microscope (FESEM) Observation

FESEM was used to observe the morphologic characteristics of the cell changes. Different volumes of citral nano-emulsion were added to the LB liquid medium to make the final concentration of citral 0, 1/4MIC, 1/2MIC, and MIC, respectively. *V. parahaemolyticus* was inoculated at 1% (*v*/*v*) and co-cultured for 9 h. Subsequently, 1.5 mL of the bacterial culture was centrifuged to pellet the cells. The pellet was resuspended and fixed in 1 mL of 2.5% (*v*/*v*) glutaraldehyde solution, followed by incubation at 4 °C for 2 h. After glutaraldehyde was fixed, it was centrifuged at 8000 r/min for 3 min, the supernatant was removed, and 1 mL of PBS was added. After shaking for 10 min, it was centrifuged at 8000 r/min for 3 min to remove the supernatant and repeated 3-fold. The bacterial was then subjected to a graded ethanol series for dehydration. It was sequentially soaked in 50%, 70%, and 90% (*v*/*v*) ethanol for 8 min per concentration, followed by centrifugation at 8000 rpm for 3 min after each step. Subsequently, dehydration was completed with absolute ethanol for 10 min. Then, the sample was treated with 1 mL of isoamyl acetate for 30 min or overnight. After the supernatant was removed, the sample was dried in an oven at 70 °C for 3 h. Finally, the dried sample was mounted on conductive tape, sputter-coated with a gold layer, and examined under FESEM (MLA650F, FEI, Hillsboro, OR, USA) for observation and imaging. 

### 2.11. Motility Assay 

The effects of citral nano-emulsion on the swarming and swimming properties of *V. parahaemolyticus* were determined with 1.5% and 0.3% (*w*/*v*) LB solid medium, respectively. The content of citral nano-emulsion in the culture medium was 0, 1/4MIC, 1/2MIC, and MIC. Afterwards, 5 μL of *V. parahaemolyticus* was placed on a solid plate and allowed to stand for 30 min. Then, it was incubated at 37 °C for 24 h (swarming) and 12 h (swimming), and the colony diameter was observed by taking photos.

### 2.12. Biofilm Formation Assay

The content of biofilm was determined by the crystal violet staining method. Briefly, the different concentrations (1/4MIC, 1/2MIC, and MIC) of citral nano-emulsion were added to LB liquid medium. *V. parahaemolyticus* of 1% (*v*/*v*) was added and incubated for 48 h. After the bacterial suspension was removed, the adherent cells were stained with 2% (*w*/*v*) crystal violet for 5 min. Subsequently, the samples were washed gently with water three times to remove unbound dye. The bound crystal violet was then eluted with 1 mL of 33% (*v*/*v*) glacial acetic acid. Finally, the absorbance of the eluent was measured at 570 nm using a spectrophotometer.

### 2.13. Extracellular Polysaccharide (EPS) Assay

The changes in EPS of *V. parahaemolyticus* were determined by the phenol sulfuric acid method. The *V. parahaemolyticus* (1%) was added to LB medium containing 1/4MIC, 1/2MIC, and MIC citral nano-emulsion. LB liquid medium without citral nano-emulsion was taken as the control group. All the above solutions were treated by a rotation speed of 200 r/min for 9 h. The supernatant was collected (8000 r/min, 5 min) and added with 3-fold the volume of 95% (*v*/*v*) ethanol. The mixed solution was deposited in the refrigerator (4 °C) for 24 h. The supernatant was discarded after centrifugation at 5000 r/min for 15 min. The obtained sediment was dissolved in 1 mL of water, mixed with 1 mL of 5% (*v*/*v*) phenol, and then reacted with 5 mL of concentrated sulfuric acid. The mixture was incubated for 20 min at room temperature. The absorbance value at 490 nm was measured.

### 2.14. Determination of Extracellular Protease

Following a 9 h exposure of *V. parahaemolyticus* to varying concentrations (0, 1/4MIC, 1/2MIC, and MIC) of the citral nano-emulsion, the optical density of each culture was measured at 600 nm. To assess protease activity, 1 mL of bacterial solution was mixed with 1 mL of PBS and 0.01 g of Hide Powder Azure (HPA, Sigma Aldrich, St. Louis, MO, USA), followed by incubation at 37 °C with shaking at 200 rpm for 2 h. After centrifugation, the supernatant was collected, and its absorbance was measured at 600 nm. Extracellular protease activity was calculated by the measured OD_600 nm_ ratio.

### 2.15. Determination of Preservation Effect of Citral Nano-Emulsion on Salmon

Salmon meat was used as a biological model to determine the preservation effect of citral nano-emulsion on aquatic products infected by *V. parahaemolyticus*. Firstly, the fresh salmon meat (sold in Xi’an) was cut into small pieces. Then, the cut salmon slices were weighed, each weighing 10 g, and moved 3 times on the flame of the alcohol lamp, 3 s each time. Next, it was irradiated with an ultraviolet lamp for 30 min, 15 min each side, for standby. Salmon slices were infected with activated *V. parahaemolyticus* (OD_600 nm_ = 0.5) by surface inoculation. Then, 10 μL of inoculant was taken each time before being distributed randomly and evenly on the surface of the salmon fillet. This was repeated 20 times to make the inoculant evenly distributed. Salmon samples were divided into 6 groups: a blank group (no treatment), a blank group of citral nano-emulsion (Tween 80 of the same concentration), a control group (inoculated bacteria solution and distilled water), an MIC group, a 2MIC group, and a 4MIC group (inoculated bacteria solution and citral nano-emulsion). The above experimental groups (except the blank group) were inoculated with 200 μL of *V. parahaemolyticus*, air dried for 15 min, and then inoculated with 200 μL of corresponding treatment sample solution. All salmon slices were stored at a constant temperature of 4 °C. Salmon slices were taken out on days 0, 3, 6, and 9, sheared, and washed with 20 mL physiological saline. The cleaned solution was gradient diluted and coated on the TCBS plate in sequence. Colony counting were performed after incubation at 37 °C for 18 h. There were three replicate experiments in each group.

### 2.16. Data Analysis

Experimental data were expressed in the form of mean ± standard deviation (n = 3). GraphPad Prism 9.5.1 and Design-Expert.V8.0.6.1 software was used for data processing, plotting the data, and statistical analysis. Unpaired *t*-test methods for multiple comparisons were performed to determine the difference among groups. A * *p* < 0.05 indicated a significant difference. ** *p* < 0.01 indicated an extremely significant difference.

## 3. Results and Discussion

### 3.1. Optimization of Synthesis Conditions of Citral Nano-Emulsion

**Figure 1:**
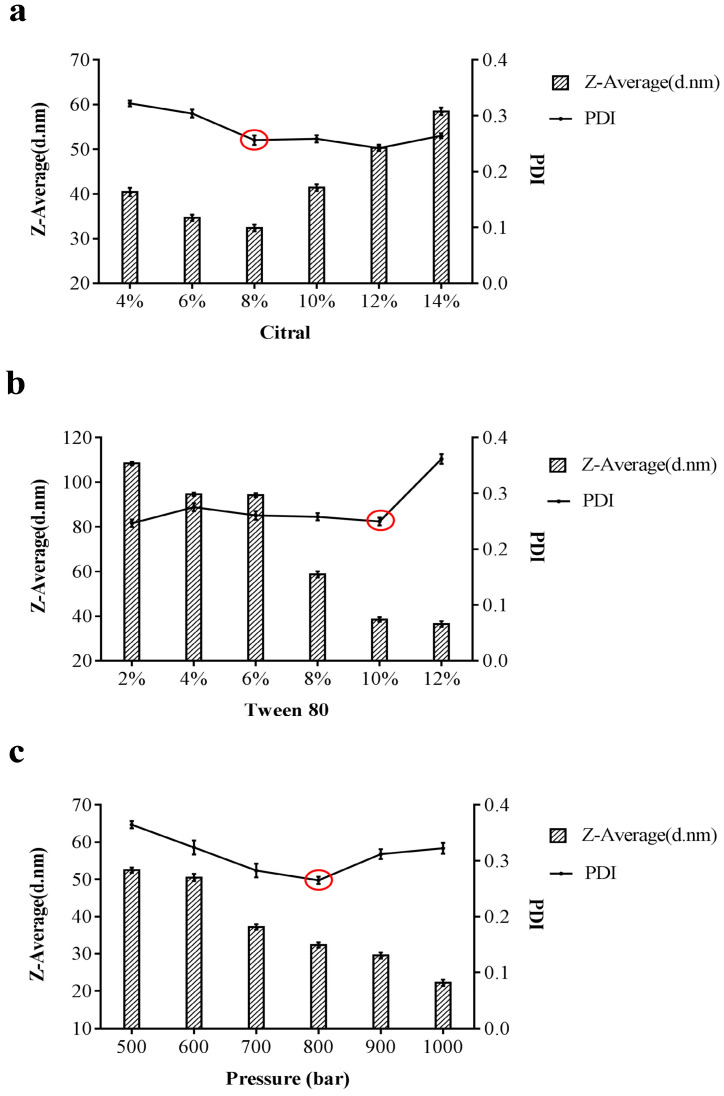
Single factor experiments on the preparation of citral nano-emulsion. (**a**) Citral content as a variable. (**b**) Tween 80 content as a variable. (**c**) Pressure as a variable. The optimal ratio, indicated by a red circle, was chosen based on the smallest particle size and PDI.

Despite having potent antibacterial properties, citral’s hydrophobicity poses a significant challenge to its application in the field of food preservation. Therefore, we proposed to prepare oil-in-water citral nano-emulsion from water-insoluble citral by high-pressure homogenization to overcome the application dilemma caused by the hydrophobic characteristics of citral. The characteristics of nano-emulsion were dependent on the properties of the emulsifier and the volume ratio of the two phases. Furthermore, the emulsifier played a crucial role in decreasing the interfacial tension between the two phases to enhance the stability of the nano-emulsion [23,24]. In general, Tween 80 was commonly employed in the food industry due to its excellent emulsifying properties [25]. In this study, Tween 80 was selected as the emulsifier of citral nano-emulsion. Since nano-emulsions were thermodynamically unstable systems, the concentration of emulsifiers was a crucial step to improve the stability of nano-emulsions [26]. In addition, the stability of the nano-emulsion system was also related to the PDI. Typically, the PDI range falls between 0 and 1. The more homogeneous the nano-emulsion’s particle size, the lower the PDI [27]. A single factor test with three factors and six levels was designed using the citral nano-emulsion’s particle size and PDI as the test indicators and citral content, Tween 80 content, and preparation pressure as the factors. As shown in Figure 1a, the nano-emulsion prepared with 8% (*v*/*v*) citral yielded the smallest particle size and a PDI of less than 0.3, leading to its selection as the optimal formulation due to its superior stability. As can be seen from Figure 1b, when Tween 80 was used as the variable, the particle size of the citral nano-emulsion prepared with a Tween 80 content of 10% (*v*/*v*) and 12% (*v*/*v*) was the smallest. However, when the content of Tween 80 was 12% (*v*/*v*), the PDI of the nano-emulsion was greater than 0.3, and the stability of the nano-emulsion system was impoverished. Therefore, 10% (*v*/*v*) of Tween 80 was selected as the optimal value. Pressure was the vital step in preparing nano-emulsion. It can be seen from the experimental results that the larger the pressure, the smaller the particle size of the nano-emulsion. Nevertheless, when the pressure was 900 bar and 1000 bar, the PDI was greater than 0.3, and the stability of the nano-emulsion was the worst. Thus, the pressure of 800 bar was selected as the optimal value. The results of univariate experiments showed that the optimal formulation parameters were 8% (*v*/*v*) citral, 10% (*v*/*v*) Tween 80, and a pressure of 800 bar. Under these conditions, the prepared citral nano-emulsion had the smallest and most uniform particle size. The experimental results above demonstrated that the ratio of citral and Tween 80 and pressure significantly affect the characteristics of the nano-emulsion. By optimizing these parameters, an ideal citral nano-emulsion with appropriate particle size and stability can be prepared, laying a robust foundation for its efficacy in practical applications.

### 3.2. Response Surface Methodology Analysis

Response surface methodology was adopted to further optimize the formulation, based on the trends observed in the single-factor experiments. Using Design-Expert software (V8.0.6.1), the experimental data were subjected to multiple regression analysis. The analysis produced a quadratic regression equation, where the particle size (Y) was expressed as a function of the independent variables:

(2) Y = 33.44 − 0.83A + 1.57B − 2.07C + 0.82AB + 3.35AC + 0.65BC + 3.37A^2^ + 5.84B^2^ + 10.89C^2^.

The established regression model was highly significant (*p* < 0.0001), the misfit term was not significant (*p* > 0.05), and the correlation coefficient of the model, R^2^ = 0.9909, and the corrected correlation coefficient, R^2^_adj_ = 0.9791, indicated that the model was well fitted and the experimental error was small (Table 2). Therefore, the model can be used to prepare citral nano-emulsion with smaller particle size.

**Figure 2:**
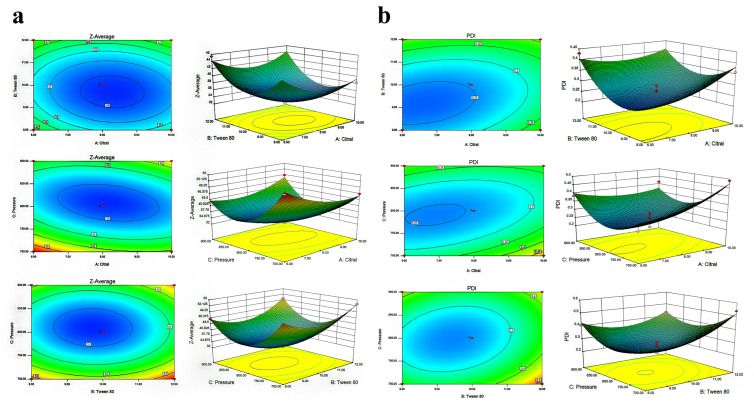
Response surface experiment on preparation of citral nano-emulsion. (**a**) Particle size of citral nano-emulsion. (**b**) PDI of citral nano-emulsion.

The response surface graph was a three-dimensional surface graph of the response surface formed by various experimental factors. From the response surface graph, the optimal parameters and their interactions can be identified. The contour plots were elliptical in shape, and the response surface showed steep slopes for the interactions between A and B, A and C, and B and C (Figure 2a). This demonstrated that these pairwise interactions were not only statistically significant but also had a large effect on the particle size. Design-Expert.V8.0.6.1 software was used to perform multiple regression fitting on the experimental results of citral nano-emulsion optimization; the quadratic regression equation with citral nano-emulsion PDI as the objective function is as follows:

(3) Y = 0.25 + 0.032A + 0.043B − 5.750 × 10^−3^C − 0.030AB − 0.045AC − 0.035BC + 0.032A^2^ + 0.078B^2^ + 0.11C^2^.

The established regression model was highly significant (*p* < 0.05), the misfit term was not significant (*p* > 0.05), and the correlation coefficient of the model, R^2^ = 0.9122, and the corrected correlation coefficient, R^2^_adj_ = 0.8741, indicated that the model was well fitted and the experimental error was small (Table 3). Thus, the model can be used to prepare more stable citral nano-emulsion.

The optimal parameters and interactions between parameters can be identified from the response surface plots. The location of the response maximum and the steepest slope on the surface (Figure 2b) suggest that the AB interaction effect was both statistically significant and stronger than the AC or BC interactions. Consequently, the PDI of the nano-emulsion was largely governed by this specific factor interaction. According to the previous optimization experiment, three repeated experiments were conducted to verify the optimal results (Table 4). The mean particle size of citral nano-emulsion was 35.21 ± 1.82 nm, and the PDI was 0.25. The prepared emulsion had a small particle size and high stability. Therefore, the optimal ratio of citral nano-emulsion was determined as follows: citral content at 8% (*v*/*v*), Tween 80 at 10% (*v*/*v*), distilled water at 82% (*v*/*v*).

The pressure, emulsifier concentration, and oil-to-water ratio of nano-emulsion were critical variables that impacted the stability and size of its particles. Existing studies also had optimized these parameters to obtain the optimal nano-emulsion. Patrignani et al. produced a stable natural antibacterial nano-emulsion through ultra-high-pressure homogenization [28]. Chong et al. used 6.09 wt% mixed surfactant (Tween 80/Span 80 (63:37, wt)) and 20 wt% glycerol as cosolvent to prepare red palm oil nano-emulsion through homogenization pressure (500 bar). Its particle size and PDI were 119.49 nm and 0.286, respectively [29]. Zhang et al., based on the phase change nano-emulsion preparation process, used Tween 80 (6.84 wt%) and Span 80 (6.70 wt%) as composite surfactants to encapsulate vitamin D_3_. The particle size of the prepared nano-emulsion was up to 391.47 ± 12.89 nm, and the polydispersity index was 0.23–0.31 [30]. Hou et al. obtained small particle size and stable cinnamon essential oil nano-emulsion by optimizing the concentrations of Tween 80 and hydroxypropyl-β-cyclodextrin [31]. The particle size and PDI of nano-emulsion prepared under different optimized conditions were also different. The citral nano-emulsion prepared in this study was simple in composition and easy to prepare, which facilitates its practical application.

### 3.3. Physicochemical Properties of Citral Nano-Emulsion

**Figure 3:**
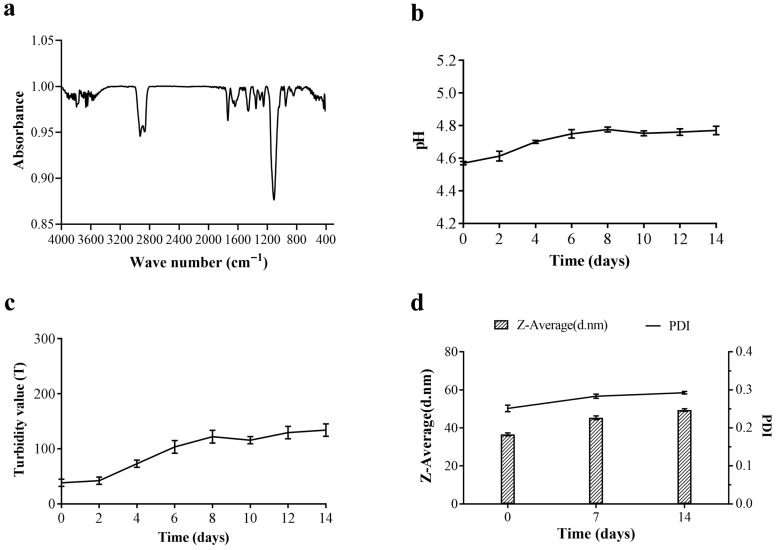
Characterization of citral nano-emulsion. (**a**) FTIR Spectra of citral nano-emulsion. (**b**) pH value of citral nano-emulsion. (**c**) Turbidity value of citral nano-emulsion. (**d**) Particle size and PDI change in citral nano-emulsion.

For better application, it is necessary to further explore the structure and stability of citral nano-emulsion. Figure 3a presents the FTIR spectrum of the citral nano-emulsion. The FTIR spectrum of the citral nano-emulsion exhibited characteristic peaks of citral at 2927 cm^−1^ and 2868 cm^−1^, which correspond to the stretching vibrations of -CH_3_ and -CH_2_-, respectively. In addition, citral nano-emulsion had a characteristic peak of aliphatic aldehyde at 1735 cm^−1^. The citral nano-emulsion had a broad and strong ether bond characteristic absorption peak around 1107 cm^−1^. The bending vibration of the C-H bond was also suggested by the faint infrared spectral peaks of citral nano-emulsion at 948 cm^−1^ and citral at 842 cm^−1^. These characteristics all included the chemical structures of Tween 80 and citral, suggesting that the two can mix to create a citral nano-emulsion. The stability of nano-emulsion was the key factor to maintaining its quality and efficacy. The changes in pH, turbidity, particle size, and PDI of citral nano-emulsion in 14 days were measured. As shown in Figure 3b, the pH of the citral nano-emulsion was kept at about 4.7, approaching 4.8 from the 6th day, and remained stable. Similarly, the turbidity of citral nano-emulsion also stabilized from the 6th day, indicating that the stability of citral nano-emulsion was excellent (Figure 3c). Additionally, the particle size and PDI of citral nano-emulsion stored at room temperature were measured as a function of storage time (Figure 3d). After 7 days, the growth rate slowed down and tended to stabilize. In the process of forming droplets in an O/W emulsion system, the newly formed droplets obtain more energy through external force, so it takes time to reach thermodynamic equilibrium. In conclusion, the citral nano-emulsion prepared under the conditions of 8% (*v*/*v*), 10% (*v*/*v*), Tween 80, and 800 bar had excellent stability.

### 3.4. Antibacterial Activity of Citral Nano-Emulsion Against V. parahaemolyticus

**Figure 4:**
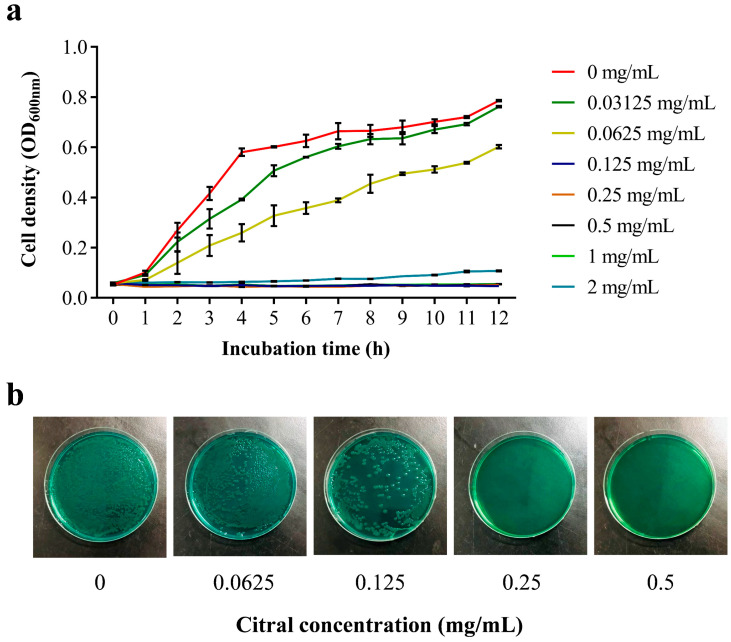
Antibacterial activity of citral nano-emulsion against *V. parahaemolyticus*. (**a**) Growth curve of citral nano-emulsion against *V. parahaemolyticus* at different concentrations. (**b**) MBC of citral nano-emulsion against *V. parahaemolyticus*.

After successfully preparing a citral nano-emulsion, the issue of citral’s insolubility in water and subsequent application impediments has been effectively resolved. To assess the antibacterial impact of citral nano-emulsion on *V. parahaemolyticus*, growth curves were plotted for *V. parahaemolyticus* RIMD 2210633 exposed to varying concentrations of citral nano-emulsion. The results of the antibacterial activity of citral nano-emulsion against *V. parahaemolyticus* by the two-fold dilution method were shown in Figure 4a. For the group treated with low-concentration citral nano-emulsion, we observed a delay in the logarithmic growth phase of *V. parahaemolyticus*, suggesting that citral nano-emulsion inhibits the initial growth of this bacterium. This inhibitory action intensifies with increasing citral nano-emulsion concentration. When the concentration of citral nano-emulsion reached 0.125 mg/mL, the growth of *V. parahaemolyticus* was inhibited. These results demonstrated that the MIC of citral nano-emulsion against *V. parahaemolyticus* was 0.125 mg/mL (based on the actual concentration of citral). The MBC of citral nano-emulsion against *V. parahaemolyticus* was determined by TCBS screening medium. As shown in Figure 4b, when the concentration of citral nano-emulsion was 0.125 mg/mL, *V. parahaemolyticus* could grow on TCBS medium, and when the concentration of citral nano-emulsion reached 0.25 mg/mL, *V. parahaemolyticus* could not grow. Therefore, the MBC of citral nano-emulsion against *V. parahaemolyticus* was 0.25 mg/mL (measured by actual concentration of citral). The results indicated that the conversion of citral into a nano-emulsion enhanced its volatility and water solubility, while the antibacterial efficacy was well preserved. This was corroborated by the consistency between the MIC and MBC values of plain citral and those of the citral nano-emulsion against *V. parahaemolyticus* [32]. The potent bactericidal properties and low concentration requirements of citral nano-emulsion highlight its advantages as a natural antibacterial agent. But not all essential oils that were made into nano-emulsions will have no effect on their antibacterial properties. After being prepared into nano-emulsion, the bacteriostatic effect of savory and thyme was similar to or lower than that of bulk oil [33]. What is more, when the essential oil extracted from Ocotea indecora’s leaves was prepared into nano-emulsion, the antibacterial concentration of nano-emulsion against *Aspergillus westerjikiae* was more than 10-fold lower than that of essential oil [34]. The emulsification process of the essential oil may result in the loss of some of its active components when it is made into nano-emulsion, hence lessening its antibacterial activity. The selection of different emulsifiers, oil–water ratios, emulsification methods, and other experimental conditions may be some factors that affect their activity. By improving the emulsification conditions, this experiment avoided the weakening of the antibacterial effect of citral nano-emulsion to the maximum extent.

### 3.5. Antibacterial Activity and Mechanism Analysis of Citral Nano-Emulsion Against V. parahaemolyticus

**Figure 5:**
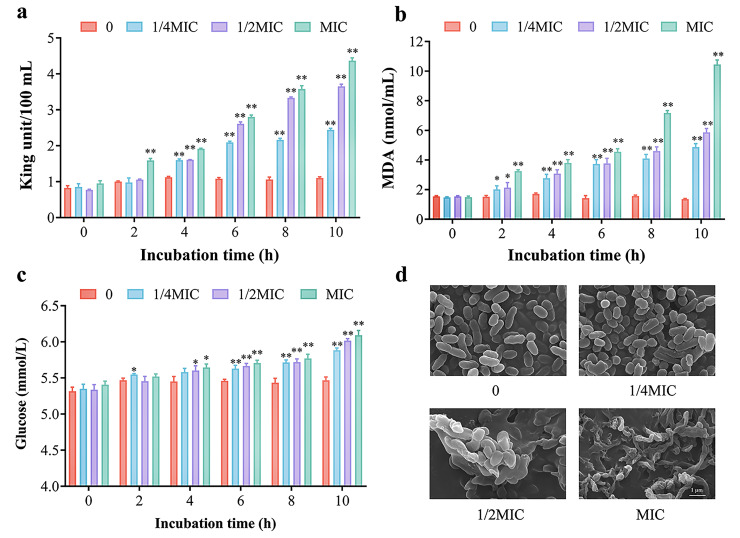
Effect of citral nano-emulsion at different concentrations on the cell membrane of *V. Parahaemolyticus*. (**a**) Effect of citral nano-emulsion on AKP of *V. parahaemolyticus*. (**b**) Effect of citral nano-emulsion on MDA of *V. parahaemolyticus*. (**c**) Effect of citral nano-emulsion on glucose of *V. parahaemolyticus*. (**d**) FESEM images showing morphological alterations of *V. parahaemolyticus* after treatment with citral nano-emulsion at different concentrations. The scale bars represent 1 μm. A * *p* < 0.05 indicated a significant difference. ** *p* < 0.01 indicated a highly significant difference.

The citral nano-emulsion demonstrated a significant inhibitory effect against *V. parahaemolyticus*. To ascertain whether the inhibitory effect results in bacterial death by disrupting its cell wall membrane, the levels of AKP, MDA, and glucose were measured in this study. AKP is an enzyme that exists between the cell wall and the cell membrane. The presence of AKP cannot be detected extracellularly when the cell structure is intact without damage. When the cell wall permeability changes, AKP leaks out of the cell [35]. As shown in Figure 5a, after the treatment of citral nano-emulsion, the AKP content in the bacterial solution gradually increased, and 0.125 mg/mL was the most significant increase (*p* < 0.01); that is, citral nano-emulsion would destroy the integrity of the cell wall of *V. parahaemolyticus*, resulting in AKP leaking out of the cell. MDA serves as the ultimate product of lipid peroxidation, reflecting the extent of oxidation in bacterial membrane lipids [36]. *V. parahaemolyticus* was treated with varying concentrations of citral nano-emulsion, and the MDA levels increased dramatically with increasing citral nano-emulsion treatment time and concentration, as determined by analysis (Figure 5b). At 10 h, MDA content increased about 6-fold after treatment with 0.125 mg/mL citral nano-emulsion compared with the control. Moreover, the extracellular glucose levels exhibited an increase in response to escalating concentrations of citral nano-emulsion, particularly noticeable after 6 h (Figure 5c). This result indicated that membrane rupture or functional impairment can cause glucose inside the cell to leak out of the cell, thereby reducing the physiological function and metabolic capacity of bacterial cells. This was comparable to the outcome of *Staphylococcus aureus* cell content leakage that occurred when Sugumar et al. utilized nano-emulsion to suppress the bacteria [37].

Through the analysis of extracellular AKP, MDA, and glucose levels, it was observed that citral nano-emulsion potentially exerts its effect by disrupting the integrity of bacterial cell membranes, leading to the leakage of these substances into the extracellular. The rupture or increased permeability of bacterial cell membranes can lead to the loss of intracellular substances, which has a fatal impact on the survival and growth of bacteria. To more intuitively determine the damage of citral nano-emulsion to the cell wall and membrane, the morphology of *V. parahaemolyticus* treated with different concentrations of citral nano-emulsion was observed by FESEM (Figure 5d). The results showed that *V. parahaemolyticus* cells not treated with citral nano-emulsion showed typical *vibrio* features, with complete cell structure and a smooth, short rod appearance. The outer membrane of *V. parahaemolyticus* treated with 1/4MIC citral nano-emulsion was missing, and a few cells were bonded together. The *V. parahaemolyticus* cells treated with 1/2MIC citral nano-emulsion mostly adhered together, the cell wall membrane was damaged, and basically no complete cell structure was found. After the treatment of MIC citral nano-emulsion, *V. parahaemolyticus* cells had a serious shrinkage phenomenon, and the shriveled and incomplete cells were all attached together, and the cell structure underwent serious changes. These results suggested that the number and degree of cell damage increased with the increase in the concentration of citral nano-emulsion, indicating that citral nano-emulsion can destroy the cell wall membrane structure of *V. parahaemolyticus* in a concentration-dependent manner. This was consistent with earlier research in which *Aeromonas hydrophila* was likewise inhibited by linalool nano-emulsion through disruption of its cell membrane [38]. Similarly, after sage oil was made into nano-emulsion, its antibacterial activity was boosted by improving its capacity to damage bacterial cell membranes [39]. In addition, *Zanthoxylum bungeanum* pericarp essential oil nano-emulsion also caused bacterial apoptosis by damaging the permeability and integrity of the cell membrane, leading to leakage of cell contents [40].

### 3.6. Effect of Citral Nano-Emulsion on Virulence of V. parahaemolyticus

**Figure 6:**
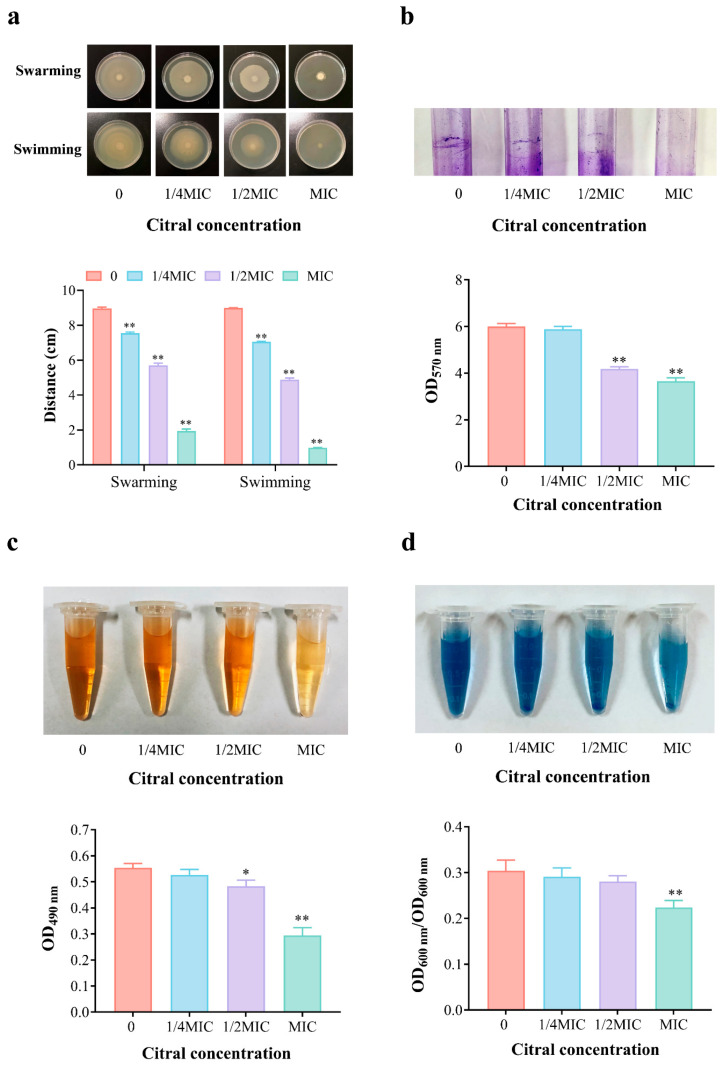
Effect of citral nano-emulsion at different concentrations on the virulence of *V. Parahaemolyticus*. (**a**) Effect of citral nano-emulsion on motility of *V. parahaemolyticus*. (**b**) Effect of citral nano-emulsion on biofilm of *V. parahaemolyticus*. (**c**) Effect of citral nano-emulsion on extracellular polysaccharide of *V. parahaemolyticus*. (**d**) Effect of citral nano-emulsion on extracellular protease of *V. parahaemolyticus*. Significance levels are indicated with asterisks: * *p* < 0.05, **p < 0.01.

The virulence of bacteria plays a crucial role in determining their pathogenicity. Therefore, we investigated the attenuation effect of citral nano-emulsion on *V. parahaemolyticus*. This mainly included changes in the motility, biofilm, EPS, and extracellular proteases of *V. parahaemolyticus* after treatment with citral nano-emulsion. The motility of bacteria is closely linked to their infectivity. As shown in Figure 6a, *V. parahaemolyticus* treated with citral nano-emulsion significantly reduced its swarming and swimming abilities. The control group bacteria showed normal motility on both hard and soft plates. In contrast, the citral nano-emulsion-treated group exhibited a significant reduction in both the swarming and swimming distances of the bacteria. Especially in the MIC group, the swarming and swimming abilities were reduced by 78.4% and 89.2%, respectively. The reduction in motility suggested that citral nano-emulsion interfered with the flagellar function of *V. parahaemolyticus*. This interference reduced the ability of bacteria to colonize and spread in host tissues, thereby diminishing their pathogenicity.

In the natural environment, over 99.9% of bacteria exist in the form of biofilms, and 65% of human bacterial infections are related to the biofilms produced by bacteria and their formation. Anti-biofilm strategies have become an essential approach for novel antimicrobial agent development. The effect of citral nano-emulsion on biofilm inhibition was evaluated at sub-inhibitory concentrations using crystal violet staining. As shown in Figure 6b, it can be observed that without treatment with citral nano-emulsion, *V. parahaemolyticus* can form a biofilm on the surface of the glass tube. After staining with 2% crystal violet and subsequent removal of free cells, a clearly visible biofilm ring can be seen on the tube wall. With the increased concentration of citral nano-emulsion, the biofilm ring gradually dissipated. Upon dissolution of the biofilm in glacial acetic acid, the absorbance of the 1/2MIC and MIC groups notably decreased at 570 nm (*p* < 0.01); in comparison to the control group, the absorbance decreased by 30.3% and 39.0%, respectively. This illustrated that citral nano-emulsion can effectively inhibit the formation of biofilms in *V. parahaemolyticus*, thereby reducing its ability to survive in the host. EPS is a significant component of bacterial biofilms, facilitating bacterial adherence and biofilm development. *V. parahaemolyticus* can increase animal immunogenicity and enhance virulence by secreting EPS, making it more difficult to control. The content of EPS measured by phenol–sulfuric acid methods also decreases with the increase of citral nano-emulsion treatment concentration (Figure 6c). Especially when the concentration reaches MIC, the EPS content significantly decreases by 46.9%. The findings suggested that citral nano-emulsion may impair bacterial biofilm formation by inhibiting the synthesis or release of EPS. The formation of biofilm was also related to motility [41]. Citral nano-emulsion reduced *V. parahaemolyticus* motility, prevented EPS synthesis, and thereby reduced biofilm formation. Previous studies have shown that eugenol and its nano-emulsion have similar inhibitory effects on the virulence factors and biofilm formation of *Pseudomonas aeruginosa* [42].

Extracellular proteases are key virulence factors of bacteria and essential tools used by various microorganisms during colonization, which can cause damage to host proteins [43]. The influence of citral nano-emulsion on the synthesis of extracellular protease of *V. parahaemolyticus* was shown in Figure 6d. The control group exhibited the highest extracellular protease activity, which decreased progressively with rising citral nano-emulsion concentrations. Among them, the MIC group showed a significant 26.3% decrease. Citral nano-emulsion significantly reduced protease activity, indicating that it can also weaken *V. parahaemolyticus* virulence by inhibiting protease synthesis. This mechanism decreases *V. parahaemolyticus* damage to host tissues, consequently lowering their infectivity. In summary, citral nano-emulsion inhibited the virulence of *V. parahaemolyticus* through various mechanisms. It significantly diminished *V. parahaemolyticus* pathogenicity by reducing motility, decreasing the formation of biofilms and extracellular polysaccharides, and inhibiting extracellular protease activity. The findings not only uncovered the anti-virulence mechanism of citral nano-emulsion but also provided theoretical support for its potential in practical applications.

### 3.7. Analysis of the Preservation Effect of Citral Nano-Emulsion on Salmon

**Figure 7:**
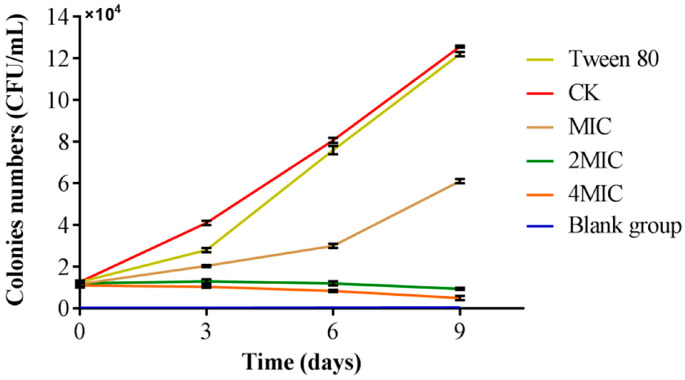
Inhibition of citral nano-emulsion on *V. parahaemolyticus* in salmon.

Salmon is a species of fish that is frequently eaten raw and is well-known for its abundance of nutrients. Nevertheless, salmon is not heated when consumed as sushi and sashimi, making it susceptible to pathogenic bacteria, especially *V. parahaemolyticus* [44]. It is possible to have nausea, vomiting, diarrhea, and other gastrointestinal symptoms after consuming tainted seafood [45]. This had to draw our attention to the infection of *V. parahaemolyticus* in salmon. The study aimed to investigate the antibacterial effects against *V. parahaemolyticus* infections on salmon treated with a high concentration of citral nano-emulsion in comparison to salmon that were not treated, as illustrated in Figure 7. When untreated with citral nano-emulsion, the amount of *V. parahaemolyticus* in salmon increased significantly. The further increase in *V. parahaemolyticus* levels after Tween 80 treatment demonstrates that this surfactant does not inhibit its growth. The growth of *V. parahaemolyticus* in salmon slices were significantly impeded when exposed to citral nano-emulsion at the MIC, resulting in a slower growth rate compared to the control group. When salmon slices were treated with citral nano-emulsion at 2MIC and 4MIC concentrations, the colonies of *V. parahaemolyticus* decreased over time as opposed to growing. Following the 9-day storage period, only a few colonies were present in both the 2MIC and 4MIC groups, indicating a favorable preservation effect. This might be the result of the essential oil’s delayed release from the nano-emulsion, which allows it to remain on the product’s surface for a longer period of time [46]. The experimental results indicated that the low concentration citral nano-emulsion exhibited a significant inhibitory effect, whereas the high concentration citral nano-emulsion demonstrated bactericidal properties, making it suitable for preserving salmon freshness. These results supported the application prospects of citral nano-emulsion as a natural preservative, especially in the fields of food safety and quality management. Future studies should evaluate the long-term stability and efficacy of the formulation. This assessment under real-world supply chain conditions, including variable temperature scenarios, is crucial for translating these laboratory findings into practical applications.

## 4. Conclusions

In conclusion, the study prepared a stable citral nano-emulsion using an optimized formulation derived from single-factor and response surface methodology, effectively overcoming the intrinsic hydrophobicity of citral and significantly improving its dispersibility and bioavailability. The citral nano-emulsion exhibited potent, concentration-dependent antibacterial effects against *V. parahaemolyticus*, primarily by disrupting cell membrane integrity and increasing permeability. It also markedly inhibited key virulence factors, including biofilm formation, motility, extracellular polysaccharide secretion, and protease activity. Notably, in salmon preservation trials, the citral nano-emulsion at elevated concentrations (2MIC and 4MIC) substantially suppressed *V. parahaemolyticus* growth, demonstrating great potential as a natural preservative. Future studies should extend these findings to other food matrices to further explore the antimicrobial potential of the nano-emulsion beyond salmon. These findings not only provide a feasible strategy for broadening the application of hydrophobic essential oils like citral in food environments but also contribute meaningfully to the development of green antibacterial agents and the enhancement of food safety. This study offers both theoretical and practical support for the use of citral nano-emulsion as a sustainable alternative to synthetic preservatives, promoting its future utilization in seafood safety.

## Figures and Tables

**Table 1:** Response surface design layout and experimental results.

Experiment Number	A Citral Content/(%)	B Tween 80 Content/(%)	C Pressure/(bar)	Mean Particle Size/(d.nm)	PDI
1	8.00	10.00	800	33.57	0.263
2	8.00	8.00	700	51.66	0.374
3	8.00	12.00	900	49.98	0.431
4	6.00	10.00	900	42.35	0.383
5	10.00	10.00	700	46.35	0.49
6	8.00	10.00	800	32.51	0.203
7	8.00	10.00	800	33.95	0.251
8	6.00	12.00	800	45.35	0.429
9	10.00	10.00	900	49.11	0.415
10	6.00	10.00	700	52.99	0.28
11	10.00	8.00	800	38.31	0.359
12	8.00	8.00	900	46.03	0.408
13	8.00	12.00	700	53.01	0.539
14	6.00	8.00	800	43.33	0.293
15	8.00	10.00	800	33.92	0.261
16	10.00	12.00	800	43.59	0.376
17	8.00	10.00	800	33.26	0.289

**Table 2:** Particle size regression analysis of citral nano-emulsion.

Source	Sum of Squares	df	Mean Square	F-Value	*p*-Value	Significance
Model	859.92	9	95.55	84.32	<0.0001	Significant
A-Citral	5.54	1	5.54	4.89	0.0626	-
B-Tween 80	19.85	1	19.85	17.51	0.0041	-
C-Pressure	34.20	1	34.20	30.18	0.0009	-
AB	2.66	1	2.66	2.34	0.1696	-
AC	44.89	1	44.89	39.62	0.0004	-
BC	1.69	1	1.69	1.49	0.2615	-
A^2^	47.72	1	47.72	42.11	0.0003	-
B^2^	143.43	1	143.43	126.58	<0.0001	-
C^2^	499.47	1	499.47	440.79	<0.0001	-
Residual	7.93	7	1.13	-	-	-
Lack of fit	6.53	3	2.18	6.20	0.0552	Not significant
Pure error	1.40	4	0.35	-	-	-
Cor total	867.86	16	-	-	-	-
R^2^ = 0.9909 R^2^_adj_ = 0.9791						

**Table 3:** Regression analysis results of PDI.

Source	Sum of Squares	df	Mean Square	F-Value	*p*-Value	Significance
Model	0.12	9	0.014	8.08	0.0058	Significant
A-Citral	8.128 × 10^−3^	1	8.128 × 10^−3^	4.75	0.0656	-
B-Tween 80	0.015	1	0.015	8.50	0.0225	-
C-Pressure	2.645 × 10^−4^	1	2.645 × 10^−4^	0.15	0.7058	-
AB	3.540 × 10^−3^	1	3.540 × 10^−3^	2.07	0.1934	-
AC	7.921 × 10^−3^	1	7.921 × 10^−3^	4.63	0.0684	-
BC	5.041 × 10^−3^	1	5.041 × 10^−3^	2.95	0.1297	-
A^2^	4.427 × 10^−3^	1	4.427 × 10^−3^	2.59	0.1517	-
B^2^	0.026	1	0.026	15.14	0.0060	-
C^2^	0.047	1	0.047	27.75	0.0012	-
Residual	0.012	7	1.710 × 10^−3^	-	-	-
Lack of fit	8.009 × 10^−3^	3	2.670 × 10^−3^	2.69	0.1812	Not significant
Pure error	3.963 × 10^−3^	4	9.908 × 10^−4^	-	-	-
Cor total	0.14	16	-	-	-	-
R^2^ = 0.9122 R^2^_adj_ = 0.8741						

**Table 4:** Repeatability test results.

Experiment Times	Particle Size/(nm) *	PDI
1	35.53 ± 1.48	0.238 ± 0.112
2	32.64 ± 2.36	0.259 ± 0.045
3	37.47 ± 1.61	0.264 ± 0.068

* Mean: 35.21 ± 1.82 nm.

## Data Availability

The original contributions presented in this study are included in the article. Further inquiries can be directed to the corresponding author.
